# 3-Hydroxykynurenine and 3-Hydroxyanthranilic Acid Enhance the Toxicity Induced by Copper in Rat Astrocyte Culture

**DOI:** 10.1155/2017/2371895

**Published:** 2017-07-31

**Authors:** Daniela Ramírez-Ortega, Alelí Ramiro-Salazar, Dinora González-Esquivel, Camilo Ríos, Benjamín Pineda, Verónica Pérez de la Cruz

**Affiliations:** ^1^Departamento de Neuroquímica, Instituto Nacional de Neurología y Neurocirugía Manuel Velasco Suárez, S.S.A, 14269 México City, MEX, Mexico; ^2^Biochemistry, Universidad Nacional Autónoma de México (UNAM), México City, MEX, Mexico; ^3^Laboratorio de Neuroinmunología, Instituto Nacional de Neurología y Neurocirugía Manuel Velasco Suárez, S.S.A, 14269 México City, MEX, Mexico

## Abstract

Copper is an integral component of various enzymes, necessary for mitochondrial respiration and other biological functions. Excess copper is related with neurodegenerative diseases as Alzheimer and is able to modify cellular redox environment, influencing its functions, signaling, and catabolic pathways. Tryptophan degradation through kynurenine pathway produces some metabolites with redox properties as 3-hydroxykynurenine (3-HK) and 3-hydroxyanthranilic acid (3-HANA). The imbalance in their production is related with some neuropathologies, where the common factors are oxidative stress, inflammation, and cell death. This study evaluated the effect of these kynurenines on the copper toxicity in astrocyte cultures. It assessed the CuSO_4_ effect, alone and in combination with 3-HK or 3-HANA on MTT reduction, ROS production, mitochondrial membrane potential (MMP), GHS levels, and cell viability in primary cultured astrocytes. Also, the chelating copper effect of 3-HK and 3-HANA was evaluated. The results showed that CuSO4 decreased MTT reduction, MMP, and GSH levels while ROS production and cell death are increasing. Coincubation with 3-HK and 3-HANA enhances the toxic effect of copper in all the markers tested except in ROS production, which was abolished by these kynurenines. Data suggest that 3-HK and 3-HANA increased copper toxicity in an independent manner to ROS production.

## 1. Introduction

Metals have a vital participation in some cellular processes as enzyme cofactors, as structural and antioxidant components, and also as part of metabolism. For this reason, their balance in cell environment is important and their unbalance causes damage in elemental cell structures as lipids and DNA; therefore, metals excess can be toxic mainly by oxidative stress production [1–3]. Copper is the third most abundant essential transition metal that is naturally found in human liver [4] and the most abundant in the brain [5]; this metal works as structural and functional part in various systems. In CNS, copper can act as cofactor of some antioxidant enzymes like + copper/zinc-dependent superoxide dismutase (SOD-1) and has big importance in respiration mitochondrial respiration as part of cytochrome c oxidase structure [6, 7]. In addition, this metal participates in the neurotransmitter biosynthesis (noradrenaline) and can be stored in ceruloplasmin [8–10]. Copper can be transported into the brain through copper transporter 1 (Ctr1) and toward inner of brain cells by the ATPase copper transporter (ATP7A) and the divalent metal transporter (DMT1) [11–15]. It has been reported that astrocytes have a great influence in cerebral copper homeostasis and they can store big amounts of this metal due to specific characteristics as vast amounts of DMT1, ferritin, metallothionein, and antioxidants as glutathione (GSH) [13, 16, 17]. However, alterations in copper metabolism have been related with neurodegenerative diseases as Alzheimer's (AD), Parkinson's (PD), Menkes (MD), and Wilson's (WD) diseases, triggering an oxidative stress state in cell environment, resulting in disturbance of energy metabolism and reactive oxygen species (ROS) production [18–21]. ROS can also stimulate endogenous pathways that can be modulated by redox environment as the kynurenine pathway (KP).

KP is the main route of tryptophan (Trp) catabolism. Trp is an essential amino acid, which can be metabolized through different pathways to form important substances as serotonin and melatonin, but more than 95% is degraded through KP [22], whose main aim is NAD^+^ production, an electron carrier and cofactor in some redox reactions [23]. KP is present in the liver, kidney, and brain of various mammals such as mice, rats, guinea pigs, rabbits, monkeys, and humans [22, 24, 25]. Along the pathway, different metabolites with neuroactive activity and/or redox properties are produced. KP is highly regulated by redox status of the cellular environment, but its metabolites can also modify this environment due to their redox properties [26]. The alteration in KP metabolite levels has been associated with aging and several neurodegenerative diseases as Huntington, Parkinson, and Alzheimer [27]. Specifically, 3-hydroxykynurenine (3-HK) and 3-hydroxyanthranilic acid (3-HANA) have been studied by various research groups, which describe controversial results. The metabolite 3-HK is found at nanomolar concentrations in CNS in normal conditions, but its levels are modified in neurodegenerative diseases. In fact, the amount increases as much as three times in Huntington's disease [28, 29]. It has been reported that 3-HK is able to induce cell death through apoptosis in brain regions as well as in cell cultures and *in vivo* experiments (with DNA fragmentation and chromatin condensation) [30–33]. In addition, it has been shown that 3-HK generates oxidative stress besides that it triggers protein aggregates in human lens and finally cataract formation because of its interaction with metals [33–35]. On the other hand, there are reports where it was observed that 3-HK (0–100 *μ*M) works as an antioxidant. In this context, 3-HK and 3-HANA were able to decrease lipid peroxidation and GSH oxidation in brain cortex homogenates [36]; in *Aldrichina grahami* homogenates, 3-HK was able to trap superoxide [37]. In other reports, it has been demonstrated that 3-HK can capture hydroxyl and peroxyl radicals [36, 38]. Recently, it was shown that 3-HK can have chelating properties with metals as ferrous and also can scavenge OH• and ONOO^−^ in chemical combinatory assays [39].

Moreover, 3-HANA has also ambiguous characteristics which cause toxicity in neuronal cultures and can produce protein damage due to its interaction with metals and with the ability to generate hydroxyl radicals through Fenton's reaction. Besides, it has been reported that 3-HANA can have uncoupling effect in oxidative phosphorylation and is able to decrease oxygen consumption-activating astrocytes and neuron death [33, 34, 40–42]. Nevertheless, 3-HANA is described as scavenger of OH• and ONOO^−^ in chemical combinatory assays and can act as a chelator of ferrous ion [36, 39]. In addition, 3-HANA can be an inflammatory and neuroprotector molecule since it induces hemeoxygenase-1 and suppresses cytokine and chemokine production stimulated by IL-1/IFN-*γ* and toll-like receptor (TLR) ligands leading to neuroprotection [43].

Due to the fact that 3-HK and 3-HANA influence the redox environment and knowing that copper can be toxic to the cell, the aim of this work was to determinate the effect of the coincubation of copper with these two kynurenine metabolites, in the toxicity induced by this metal.

## 2. Materials and Methods

3-hydroxykynurenine (3-HK), 3-hydroxyanthranilic acid (3-HANA), copper sulfate (CuSO_4_), thiazolyl blue tetrazolium bromide, 2′,7′-diclorodihidrofluoresceine diacetate (DCF-DA), and propidium iodide (PI) were obtained from Sigma Chemical Company (St. Louis, MO, USA). Dulbecco's modified eagle's medium (DMEM) and fetal bovine serum (FBS) were purchased from Gibco BRL (Grand Island, NY). All other chemicals were of the highest commercially available purity and obtained from known commercial suppliers. Solutions were prepared using deionized water obtained from a Milli-RQ (Millipore) purifier system.

### 2.1. Copper Chelation Assays

Chelation capacity of both KP metabolites was assessed according to previous report [44], where different concentrations of 3-HK and 3-HANA (0-1 mM) were tested. Briefly, a solution of chelator (50 *μ*l of 3-HK or 3-HANA in different concentrations) was mixed with CuSO_4_ (50 *μ*l) in HEPES buffer (50 *μ*l). After 2 minutes, 50 *μ*l of hematoxylin or DMSO (blank) was added and mixed for 3 minutes. Then, the absorbance was measured during 4 min. The wavelength used was different for each pH tested. Three different pH (5.5, 6.8, and 7.5) and 2 different buffers were tested (sodium acetate buffer pH 5.5 and HEPES buffer pH 6.8 and 7.5), considering previous reports where it was demonstrated that copper accumulation, as in the pathologies, can change pH environment [45, 46].

### 2.2. Primary Astrocyte Cultures

Rat-cultured cortical astrocytes were obtained from the brains of 3 days postnatal Wistar rats (PND). Cells were seeded in Roux flasks at a 9 × 10^6^ cells/ml density. The cells were maintained in DMEM supplemented with FBS at 10% under incubation at 37°C with CO_2_ (5%), until the cells were again seeded in 24-well plates to be used. Over 95% of the cells were immunoreactive for glial fibrillary acidic protein, an astrocyte-specific marker [39].

### 2.3. MTT Reduction Assay

According to previous reports [39, 47, 48], cellular function was evaluated by MTT reduction assay. This assay is employed as a functional status test through the formation of formazan salts by the action of dehydrogenases in viable cells [39]. Briefly, astrocytes (100,000 per well) were treated with different copper concentrations (0–500 *μ*M), to stablish the toxic copper concentration. Then, CuSO_4_ (350 *μ*M) was coincubated with 3-HK and 3-HANA (100 *μ*M) in DMEM medium for 24 h at 37°C. After treatment, the medium was removed and 500 *μ*l MTT (1 mg/ml in DMEM medium) was added to each well. MTT was incubated for 3 h at 37°C, then medium was removed, and acid isopropanol was added to dissolve the blue formazan salts. Quantification of resulting blue formazan salts was done at a wavelength of 570 nm in a plate reader (EON, BioTek). The results were expressed as the percentage of MTT reduction versus control values.

### 2.4. ROS Production Determination

ROS were evaluated through DCF-DA oxidation [49]. Astrocytes (100,000 per well) were treated with different copper concentrations (0–500 *μ*M), and then copper (350 *μ*M) was coincubated with 3-HK and 3-HANA (100 *μ*M) in DMEM medium for 24 h at 37°C. After that, medium was removed, and cells were washed with saline solution and were added with 75 *μ*l of trypsin. Cells were recollected, and 100 *μ*l of DCF-DA (75 *μ*M) was added to the tubes and reincubated for 20 min at 37°C in darkness. After incubation, ROS formation was quantified by flow cytometry at 488 nm excitation and 532 emission considering 10,000 total events in FlowJo programm. Data are presented as percentage of ROS production versus control.

### 2.5. Mitochondrial Membrane Potential (MMP) Assay

Mitochondrial membrane potential is a marker of healthy cells, and JC-1, a lipophilic cation, is used to evaluate because it is selective to changes in mitochondrial membrane potential and can form red fluorescence aggregates (FL-1 channel, emission length 525 nm) with high MMP, whereas when MMP is low, JC-1 is in its monomeric form (FL-2 channel, emission length at 590 nm) and displays a green fluorescence [50, 51]. After treatments, medium was removed, cells were washed with saline solution, and then 75 *μ*l of trypsin was added to each well. Cells were recollected and centrifuged at 2000 rpm for 10 min. Medium was discarded, and mitochondrial membrane potential was evaluated through the label of cells with 3 *μ*M of 5,5′,6,6′-tetrachloro-1,1′,3,3′-tetraethylbenzimi-dazolylcarbocyanine iodide (JC-1) for 15 min at 37°C in darkness. Then, cells were washed with buffer assay two times. After washing, cells were resuspended and analyzed by flow cytometry. 10,000 events were assessed. Data are expressed as mean fluorescence intensity (MFI) in FL-2 channel and the percentage of cells that decreased MMP [39].

### 2.6. GSH Determination

GSH concentration was measured with a glutathione detection assay kit (Abcam 65322). Briefly, astrocytes were incubated with copper (350 *μ*M), 3-HK and 3-HANA (100 *μ*M), and with combinations of both in DMEM medium for 24 h at 37°C. After that, medium was removed, cells were washed with saline solution, and then 75 *μ*l trypsin was added to each well. Cells were recollected (100,000 cells) and centrifuged at 2000 rpm for 10 min. Medium was discarded, cells were washed with cold PBS, resuspended in cell lysis buffer, homogenized, and centrifuged 10 min at 4°C, and supernatant was collected. The cells were deproteinized with perchloric acid and potassium hydroxide. Once deproteinized, samples were ready to use in the GSH determination assay according to the kit's instructions. Briefly, standard curve was prepared from 0.1 *μ*g/*μ*l of GSH and dilutions were done in lysis buffer. 50 *μ*l of standard and 100 *μ*l of each sample were added to each well, and then 2 *μ*l of GST reagent and 2 *μ*l of monochlorobimane (MCB) were added. The plate was mixed, and fluorescence in samples was immediately measured in a plate reader at 360 nm excitation and 460 nm emission in a kinetic mode, every 3 minutes for 1 hour at 37°C. The results were expressed as the percentage change in glutathione levels in treated versus untreated control samples.

### 2.7. Viability Test Assay

Cellular death was assessed with propidium iodide according to Magana-Maldonado et al. [52]. Astrocytes were incubated with the combinations of CuSO_4_ (350 *μ*M) with 3-HK or 3-HANA (100 *μ*M) in DMEM medium for 24 h at 37°C. Then, medium was removed, cells were washed with saline solution, and trypsin (75 *μ*l) was added to each well. Cells were recollected and centrifuged at 2000 rpm for 10 min. Medium was discarded, and propidium iodide (PI) was added (5 *μ*g/ml) and incubated for 15 min in darkness. After incubation with PI, samples were analyzed by flow cytometry, and a total of 10,000 events were assessed. PI fluorescence was determinated with a FACSCalibur instrument, and data collection was performed using unstained cells and positive controls for single color. The results were expressed as cells death percentage.

### 2.8. Data Analysis

The results were expressed as mean values ± SEM. All data were analyzed by one-way analysis of variance and Tukey's post hoc test using the Prism software (GraphPad, San Diego, CA, USA). Values of *p* < 0.05 were considered statistically significant.

## 3. Results

### 3.1. Concentration-Response Effects of Copper Toxicity in Astrocyte Cultures

**Figure 1:**
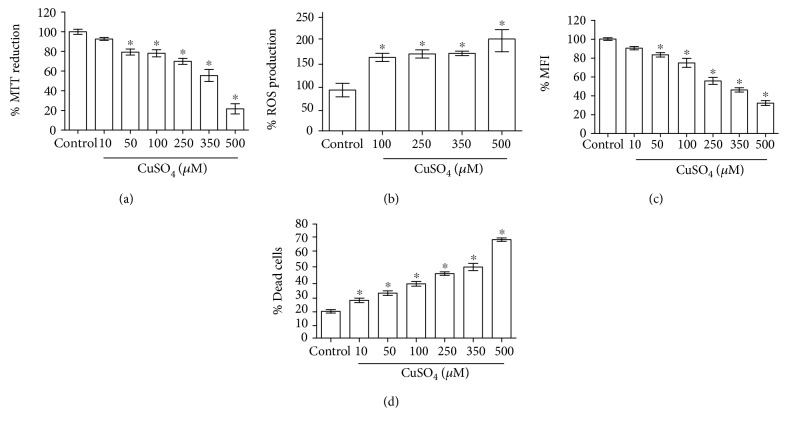
Effect of CuSO_4_ on cellular function (a), ROS production (b), MMP (c), and cell viability (d) in astrocytes. After incubation for 24 h with copper, MTT, DCFC-DA, JC-1, and iodide propidium were added to each well, respectively. Data are presented as mean values + SEM of 8 independent experiments from 4 different cultures. ^∗^*p* < 0.05 versus control (one-way ANOVA followed by Tukey's test).

To evaluate copper toxicity, astrocytes were incubated during 24 h with different copper concentrations (0–500 *μ*M) in DMEM medium. Astrocytes showed decrease on MTT reduction in a concentration-dependent manner in all used concentrations, being the major effect (80%) with 500 *μ*M of copper (Figure 1(a)). After it was proven that copper had effect on cellular function and knowing that the dehydrogenases are responsible of MTT reduction, we evaluated how mitochondrial function was affected and if ROS were implied in copper toxicity on astrocytes. Copper reduced MMP in a concentration-dependent manner (Figure 1(c)) and increased ROS production around 50% versus control; however, this effect was not concentration dependent (Figure 1(b)). After the toxicity pattern was observed, we evaluated cell death through propidium iodide (PI), which is capable of binding and labeling DNA. After incubation during 24 h with different concentrations of copper, the cell death increased significantly since the lower concentration (10 uM) was tested and this effect was concentration dependent (Figure 1(d)).

### 3.2. Copper-Chelating Ability of 3-HK and 3-HANA

**Figure 2:**
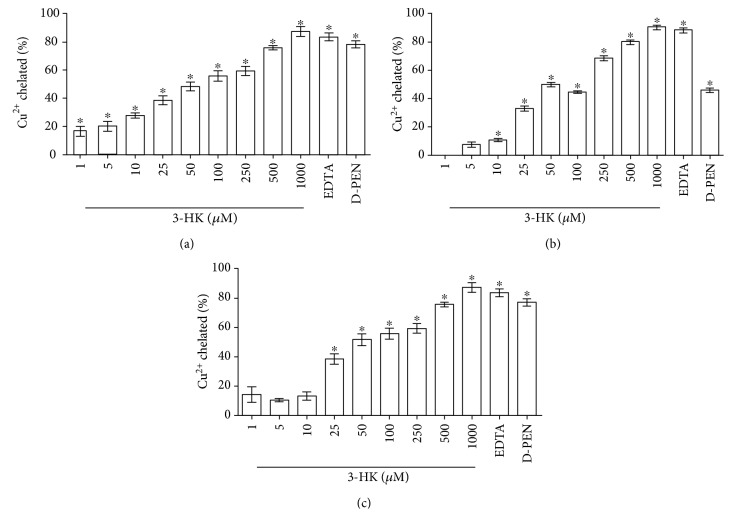
Copper chelation capacity of 3-HK. Different conditions of pH were tested: pH 5.5 (a), pH 6.8 (b), and pH 7.5 (c). Data are presented as mean values + SEM of 8 independent experiments for each concentration. ^∗^*p* < 0.001 versus control (one-way ANOVA followed by Tukey's test).

With the purpose of testing if copper had an interaction with 3-HK or 3-HANA, we assessed the chelation capacity of 3-HK and 3-HANA for copper. The assay was carried out at three different pH (5.5, 6.8, and 7.5) since we knew that copper is able to modify the pH.  Figure 2() shows the 3-HK ability to form a chelating complex with copper in all pH tested. This 3-HK ability to catch copper was more efficient in acid pH being that the IC50 is at pH 5.5 = 56.223 ± 10.322 *μ*M, IC50 at pH 6.8 = 74.731 ± 9.3231 *μ*M, and IC50 at pH 7.5 = 74.232 ± 16.769 *μ*M.

**Figure 3:**
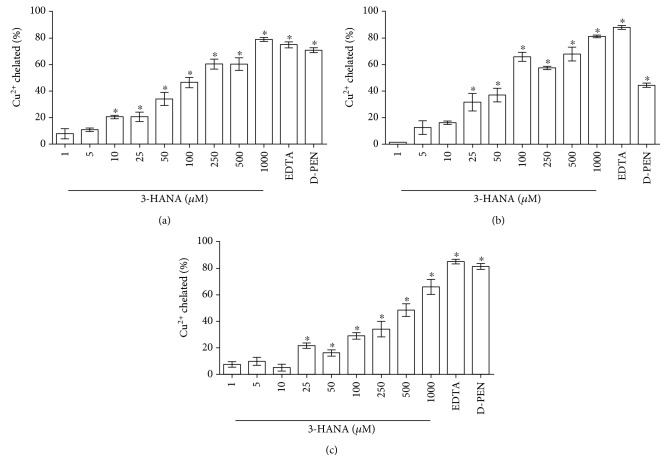
Copper chelation capacity of 3-HANA. Different conditions of pH were tested. pH 5.5 (a), pH 6.8 (b), and pH 7.5 (c). Data are presented as mean values + SEM of 8 independent experiments for each concentration. ^∗^*p* < 0.001 versus control (one-way ANOVA followed by Tukey's test).

On the other hand, 3-HANA had chelating capacity for copper too, three different conditions were tested, and in all of them, 3-HANA was able to catch copper (Figure 3). At pH 6.8, the most efficient ability to chelate copper with an IC50 = 112.491 ± 7.212 *μ*M took place, following at pH 5.5 with an IC50 = 146.637 ± 4.922 *μ*M and the pH where it was observed less effect was at pH 7.5 with at IC50 = 559.497 ± 31.422 *μ*M.

### 3.3. Effect of 3-HK and 3-HANA in the Cellular Dysfunction Induced by Copper in Astrocytes

**Figure 4:**
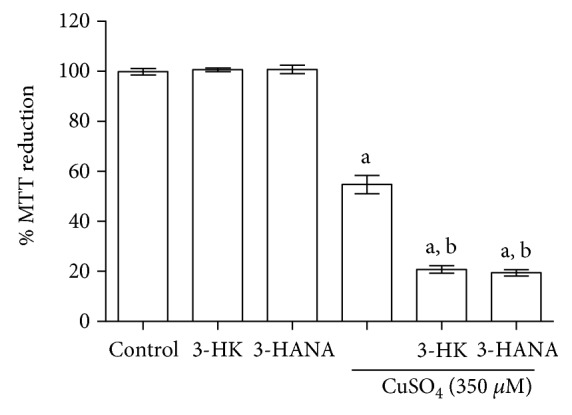
Effect of 3-HK and 3-HANA in the cellular dysfunction induced by CuSO_4_ (350 *μ*M). After 24 h of incubation with 3-HK or 3-HANA (100 *μ*M) + copper, MTT was added to each well and formazan salt was measured. Data are presented as mean values + SEM of 6 independent experiments from 3 different cultures. ^a^*p* < 0.001 versus control and ^b^*p* < 0.001 versus CuSO_4_ (one-way ANOVA followed by Tukey's test).

After copper toxicity was evaluated, we determined the kynurenine effect in the presence of this metal (350 *μ*M).  Figure 4 shows that copper decreases cellular function (around 50% versus control), evaluated by MTT reduction assay, and the coincubation with the kynurenines enhances this effect (around 80% versus control). The kynurenines alone do not have effect on MTT reduction.

### 3.4. Kynurenines Enhance the Reduction of Mitochondrial Membrane Potential Induced by Copper on Astrocytes

**Figure 5:**
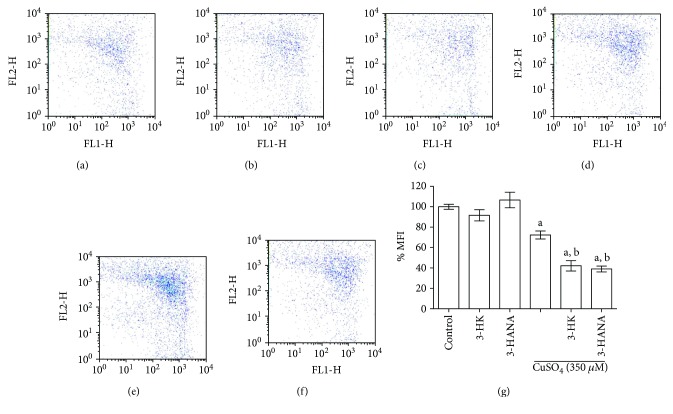
Effect of coincubation of CuSO_4_ (350 *μ*M) with 3-HK (100 *μ*M) and 3-HANA (100 *μ*M) on MMP in astrocytes. The MMP was measured using JC-1 orange-red fluorescence. Changes in MMP were evaluated by flow cytometry. Representative dot plots of MMP are showed in (a) control, (b) 3-HK, (c) 3-HANA, (d) copper, (e) copper + 3-HK, and (f) copper + 3-HANA. Percentage of mean fluorescence intensity (MFI) is present in (g). Data are presented as mean values + SEM of 6 independent experiments from 3 different cultures. ^a^*p* < 0.001 versus control and ^b^*p* < 0.001 versus CuSO_4_ (one-way ANOVA followed by Tukey's test).

The next step was to determine whether the effect on MTT reduction could be related with mitochondrial membrane potential alterations. Representative figures are shown in Figures 5(a), 5(b), 5(c), 5(d), 5(e), and 5(f). 3-HK and 3-HANA (100 *μ*M) alone do not induce effect on this parameter compared with the control group. However, copper (350 *μ*M) is able to reduce around 40% the MMP, while the coincubation of copper with both kynurenines reduced around 60% the MMP versus control.

### 3.5. 3-HK and 3-HANA Reduce ROS Production Induced by Copper

**Figure 6:**
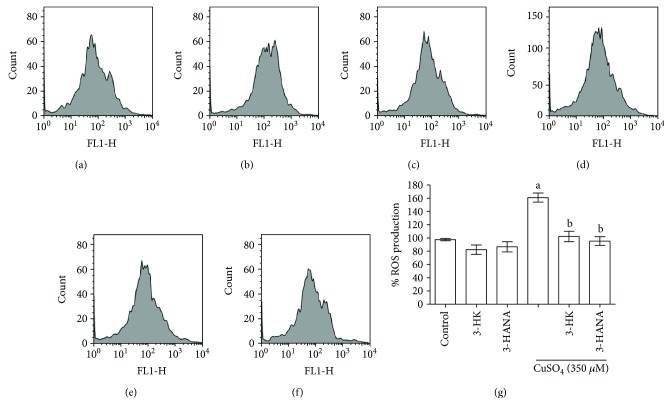
Effect of 3-HK (100 *μ*M) and 3-HANA (100 *μ*M) on ROS production induced by copper. After 24 h of incubation with the kynurenines and copper, DCF-DA was added to all treatments to determinate ROS. Representative dot plots of MMP are showed in (a) control, (b) 3-HK, (c) 3-HANA, (d) copper, (e) copper + 3-HK, and (f) copper + 3-HANA. Percentage of ROS production is showed in (g). Data are presented as mean + SEM of 6 independent experiments from 3 different cultures. ^a^*p* < 0.01 versus control and ^b^*p* < 0.001 versus CuSO_4_ (one-way ANOVA followed by Tukey's test).

Considering redox properties of 3-HK and 3-HANA, the next experiment was to know if the potentiation in the copper toxicity induced by the kynurenines was through ROS production. Copper (350 *μ*M) induces around 60% ROS production, and coincubation with 3-HK and 3-HANA abolished this effect. Incubation of 3-HK and 3-HANA alone did not have effect in ROS production (Figure 6). Representative pictures are shown in Figures 6(a), 6(b), 6(c), and 6(d).

### 3.6. GSH Depletion Is Involved in Toxicity Pattern Induced by the Coincubation of Copper and Kynurenines

**Figure 7:**
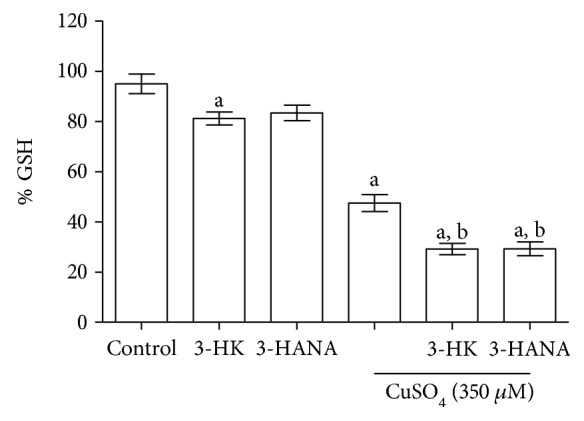
Effect of 3-HK and 3-HANA and their coincubation with copper in GSH levels on astrocytes. GSH levels were determinated after 24 h of incubation with the treatments. Data are presented as mean values + SEM of 8 independent experiments for each treatment. ^a^*p* < 0.05 versus control and ^b^*p* < 0.001 versus CuSO_4_ (one-way ANOVA followed by Tukey's test).

As we know, both copper and kynurenines can interact with GSH, and then we evaluated the levels of this endogenous antioxidant that it is in high concentration in astrocytes. 3-HK decreased GSH levels around 20%, while copper decreased them around 55%; 3-HANA did not have effect in this parameter. However, the coincubation of copper with these kynurenines decreased GSH levels around 70% versus control, in both cases (Figure 7).

### 3.7. Effect of Copper Coincubation with Kynurenines on Cell Viability

**Figure 8:**
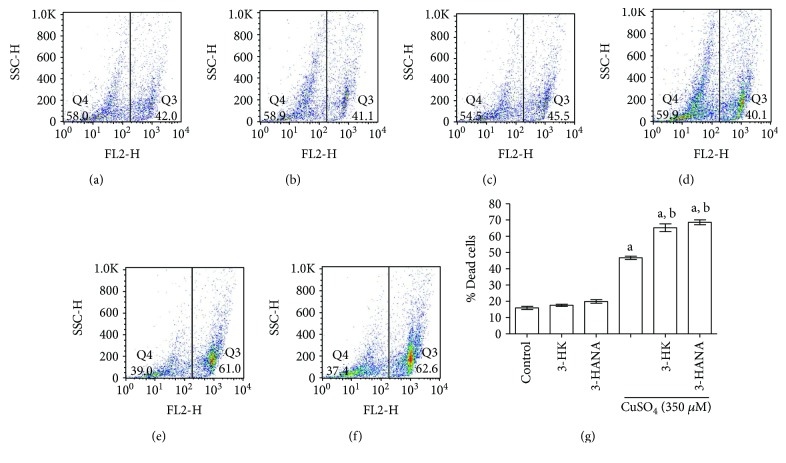
Effect of 3-HK and 3-HANA on cell death induced by copper. The propidium iodide (PI) flow cytometry assay was used for the evaluation of cell viability. Representative dot plots of IP are showed in (a) control, (b) 3-HK, (c) 3-HANA, d) copper, (e) copper + 3-HK, and (f) copper + 3-HANA. Percentage of dead cell is showed in g. Data are presented as mean values + SEM of 6 independent experiments from 3 different cultures. ^a^*p* < 0.001 versus control and ^b^*p* < 0.001 versus CuSO_4_ (one-way ANOVA followed by Tukey's test).

**Figure 9:**
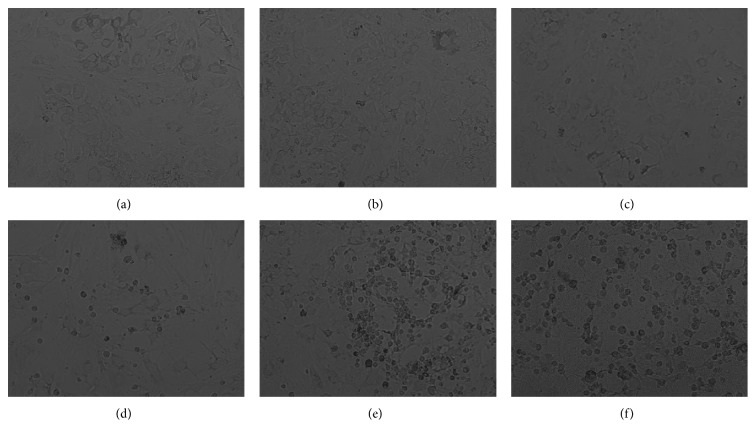
Effect of kynurenines on cell death induced by copper in astrocytes. Representative phase-contrast micrographs showing the effect of coincubation of 3-HK or 3-HANA with copper. (a) Control, (b) 3-HK, (c) 3-HANA, (d) CuSO_4_, (e) CuSO_4_ + 3-HK and (f) CuSO_4_ + 3-HANA.

Figure 8() shows the effect of copper and kynurenines on cell viability. Representative pictures are shown in Figures 8(a), 8(b), 8(c), 8(d), 8(e), and 8(f). 3-HK and 3-HANA alone did not have effect on cell death, while copper was able to increase the percentage of dead cells around 45%. Coincubation of copper with kynurenines enhanced the number of dead cells (Figure 8(g)).  Figure 9() shows representative bright field micrographs of the different treatments. 3-HK and 3-HANA did not show difference compared with control. However, in copper alone (Figure 9(d)) and the copper combination with 3-HK (Figure 9(e)) and 3-HANA (Figure 9(f)), a considerable number of dead cells compared with control can be seen.

## 4. Discussion

3-HK and 3-HANA are metabolites of tryptophan catabolism, which possess redox properties and have been associated with neurodegenerative diseases as HD and AD. These kynurenines are produced through KP, which is highly regulated by redox environment. In this context, copper is an integral part of many important enzymes involved in cellular metabolism; however, its dyshomeostasis can generate oxidative stress and it has been related with some neurodegenerative diseases in which also KP metabolites are involved [53, 54]. In the present work, we evaluated the effect of 3-HK and 3-HANA in the copper toxicity on astrocytes. We performed the experiments in astrocytes since in the brain, these cells are thought to play a key role in copper homeostasis; in fact, it has been proposed that astrocytes can normally accumulate this metal which will be used by themselves or routed to neurons [53], and the second reason to use astrocytes is that 3-HK and 3-HANA cannot be enzymatically degraded in these cells. First, we demonstrate that copper had toxic effects on astrocytes as some previous reports showed [55–57]. Copper was able to decrease cell functionality and MMP and increase ROS production; these factors may be closely related and be dependent on each other. Despite the fact that copper has important functions as cofactor of antioxidant enzymes as SOD 1, copper is also cytotoxic considering that it can participate in ROS production through Fenton's reaction and to displace other elemental metals [58, 59]. Besides, mitochondrial alterations and changes in redox environment induced by copper can lead to decrease cell viability as was observed in this work.

On the other hand, it has been shown that 3-HK and 3-HANA are able to scavenge hydroxyl radical and peroxynitrite in chemical combinatory assays and also are able to chelate some metals such as iron [39]. Keeping in mind this background, we explore if 3-HK and 3-HANA would have copper-chelating capability. Copper-chelating probes were placed at three different pH (5.5, 6.8, and 7.5) by two reasons: (1) in the method that we are using to determinate copper chelation, the affinity of hematoxylin for cupric ions is decreased when the pH is also reduced, and mainly (2) because it has been described in previous reports that copper accumulation, as in some pathologies, can change environment pH and this changes can influence the chelating capacity of various molecules [44–46]. Our data show that both kynurenine metabolites were able to chelate copper under different conditions of pH; this results may be due to the nature of 3-HK and 3-HANA since it has been reported that these metabolites are good electron donors in electrochemical experiments [60].

According to redox and chelating properties of 3-HK and 3-HANA, we decided to evaluate their effect on the copper toxicity. Both kynurenines were able to abolish ROS production induced by copper; however, the toxic effect on mitochondrial and cellular function was enhanced by the coincubation of copper with both kynurenines. These effects may be due to the fact that both 3-HK and 3-HANA are able to affect respiratory control (oxygen consumption in states 2 and 3 of mitochondrial respiration) [61] in addition to copper toxicity in mitochondria. Both sceneries affect ATP production and can lead to cell death as can be observed in Figure 9(). In this context, a previous report showed that 3-HK and 3-HANA produced cellular damage but in an independent way of ROS production, and actually, the oxidative stress parameters evaluated were even below of basal levels [39]. Moreover, the toxic copper effect enhanced by the kynurenines can be due to the fact that these kynurenines can be oxidized in the presence of copper as copper is reduced, promoting cross-linking in important proteins [34]. Besides, autoxidation of these hydroxykynurenines can form compounds as xanthommatin radical, p-quinone, and 4,6-dihydroxyquinolinequinonecarboxylic acid (DHQCA) which are reactive [62] and can interfere with the mitochondrial function and subsequently provoke cell toxicity. Other parameter evaluated was the effect of copper and kynurenines on GSH, which is in high amounts in astrocytes [17]. GSH is responsible to form complexes with copper being copper a natural pool in astrocytes; this complex is required for the incorporation of copper into metallothionein and for SOD activation. In the case of copper was overmuch, GSH would be the first antioxidant to catch it and avoid a triggering of oxidative effects [63–65]. Our data show that copper (350 *μ*M) decreased GSH levels and this effect was also enhanced by 3-HK and 3-HANA. This could be explained by the fact that in astrocytes, 3-HK can suffer a deamination and this could occur slowly at physiological pH or by the action of kynurenine aminotransferase (KAT), forming 3-hydroxykynurenine glucoside (3-OHKG) in a nonoxidative way, which in turn can form adducts with GSH [66–68]. With the knowledge that KAT is the most abundant KP enzyme in astrocytes, it is not hard to think that 3-HK deamination is taking place and 3-OHKG can form adducts with the large amounts of GSH in this cell type, decreasing GSH available to catch copper and allowing that free copper causes the greater damage observed. Although that process has not been described with 3-HANA, it has been shown that this metabolite is also able to decrease GSH levels in some kind of cells [69]. It is important to take into consideration that 3-HK and 3-HANA could be good ROS scavengers and chelating agents; however, their interaction with other cellular components could increase the cell vulnerability to damage toward other agents, as in this case, to copper.

## 5. Conclusion

This research provides important evidence about how two endogen KP metabolites can intensify the cellular damage induced by copper. It is relevant, because in some neurodegenerative diseases, they are found as common factors, the alteration in copper concentrations or in other metals, as well as KP metabolite alterations. The challenge for the future research would be to know the precise modulation of KP metabolites by metals and try to identify therapeutic targets in diseases where these components are present.
